# Survival analysis and individualized prediction of survival benefit for pancreatic signet ring cell carcinoma: a population study based on the SEER database

**DOI:** 10.1186/s12876-023-02650-5

**Published:** 2023-03-09

**Authors:** Duorui Nie, Jing Yang, Hao Zheng, Guihua Lai, Fei Wang, Jianxiong Cao, Chun Gong

**Affiliations:** 1 Department of Oncology, The First Hospital of Hunan University of Chinese Medicine, Changsha, China; 2 First Clinical Medical College, Guangzhou University of Chinese Medicine, Guangzhou, China; 3 School of Continuing Education, Hunan University of Chinese Medicine, Changsha, China; 4 Department of Oncology, The First Hospital of Hunan University of Chinese Medicine, Changsha, China

**Keywords:** Nomogram, Pancreatic signet ring cell carcinoma, Prognosis, Survival, SEER

## Abstract

**Objectives:**

This study aimed to compare the incidence, clinicopathological characteristics and survival results of pancreatic signet ring cell carcinoma (PSRCC) and pancreatic adenocarcinomas (PDAC), as well as to analyze the clinical characteristics related to the overall survival (OS) of PSRCC, and to establish an effective prognostic nomogram to predict the risks associated with patient outcomes.

**Methods:**

A total of 85,288 eligible patients including 425 PSRCC and 84,863 PDAC cases were retrieved from the Surveillance, Epidemiology, and End Results database. The survival curve was calculated using the Kaplan–Meier method and differences in them were measured by Log-rank tests. The Cox proportional hazards regression model was used to identify independent predictors of OS in patients with PSRCC. A nomogram was constructed to predict 1-, 3-, and 5-year OS. The performance of the nomogram was measured by C-index, receiver operating characteristic (ROC) curve, decision curve analysis (DCA).

**Results:**

The incidence of PSRCC is much lower than that of PDAC (10.798 V.S. 0.349 per millions). PSRCC is an independent predictor of pancreatic cancer with a poorer histological grade, a higher rate of lymph node and distant metastasis, and a poorer prognosis. We identified four independent prognostic factors including grade, American Joint Committee on Cancer Tumor-Node-Metastasis (TNM) stage, surgery and chemotherapy based on the Cox regression model. The C-index and DCA curves showed better performance of the nomogram than TNM stage. ROC curve analysis also showed that the nomogram had good discrimination, with an area under the curve of 0.840, 0.896, and 0.923 for 1-, 3-, and 5-year survival. The calibration curves showed good agreement between the prediction by the nomogram and actual observations.

**Conclusion:**

PSRCC is a rare but fatal subtype of pancreatic cancer. The constructed nomogram in this study accurately predicted the prognosis of PSRCC, performed better than the TNM stage.

## Introduction

Primary pancreatic signet ring cell carcinoma (PSRCC) is a scarce histopathological variation with an estimated incidence of less than 1% of pancreatic adenocarcinomas (PDAC) [1], which refers to the intra-cytoplasmic mucin vacuoles of more than 50% of mucin protein and the nucleus is squeezed sideways by the mucin to form a “signet ring” appearance [2]. It is reported that the 5-year overall survival (OS) of PSRCC is lower than that of pancreatic adenocarcinomas, and by the time of diagnosis, it is more prone to distant metastasis (69.4% vs. 52%) [3]. Furthermore, recent literature [4–6], also indicates that signet ring cell carcinoma (SRCC) is an independent prognostic factor for esophageal cancer, colon cancer and bladder cancer, with a worse prognosis. These findings seem to suggest that PSRCC may also have poorer histological behavior and prognosis and may require more aggressive treatment strategies. However, due to the rarity of PSRCC, the current literature mainly focuses on case reports [7–13]. The independent prognostic effect of SRCC in PDAC has not been determined in the existing literature. Although Patel et al.'s [3] study described in detail the clinical features and prognostic factors of patients with PSRCC, it did not compare them to those with PDAC and also failed to assess the role of chemotherapy and American Joint Committee on Cancer (AJCC) Tumor-Node-Metastasis (TNM) staging. To date, no large population-based study has compared the clinical features, treatment and prognosis of PSRCC and PDAC. Furthermore, specific age-adjusted incidence based on SRCC has not been identified.

In addition, nomograms have been recognized as useful tools for assessing diagnosis and prognosis by integrating important clinicopathological features [14, 15]. Compared with the traditional TNM staging system, nomograms can improve the accuracy of prognosis prediction and achieve more appropriate clinical decision making [16, 17]. However, there are no nomograms available for PSRCC at the moment. Therefore, we conducted our study based on a large and widely recognized SEER database. The research aimed to compare the clinicopathological features and survival results of PSRCC and PDAC, as well as to analyze the clinical characteristics related to the overall survival (OS) of PSRCC, and to construct and verify a nomogram prediction model unique to signet ring cell carcinoma.

## Materials and methods

### Study population

**Fig. 1:**
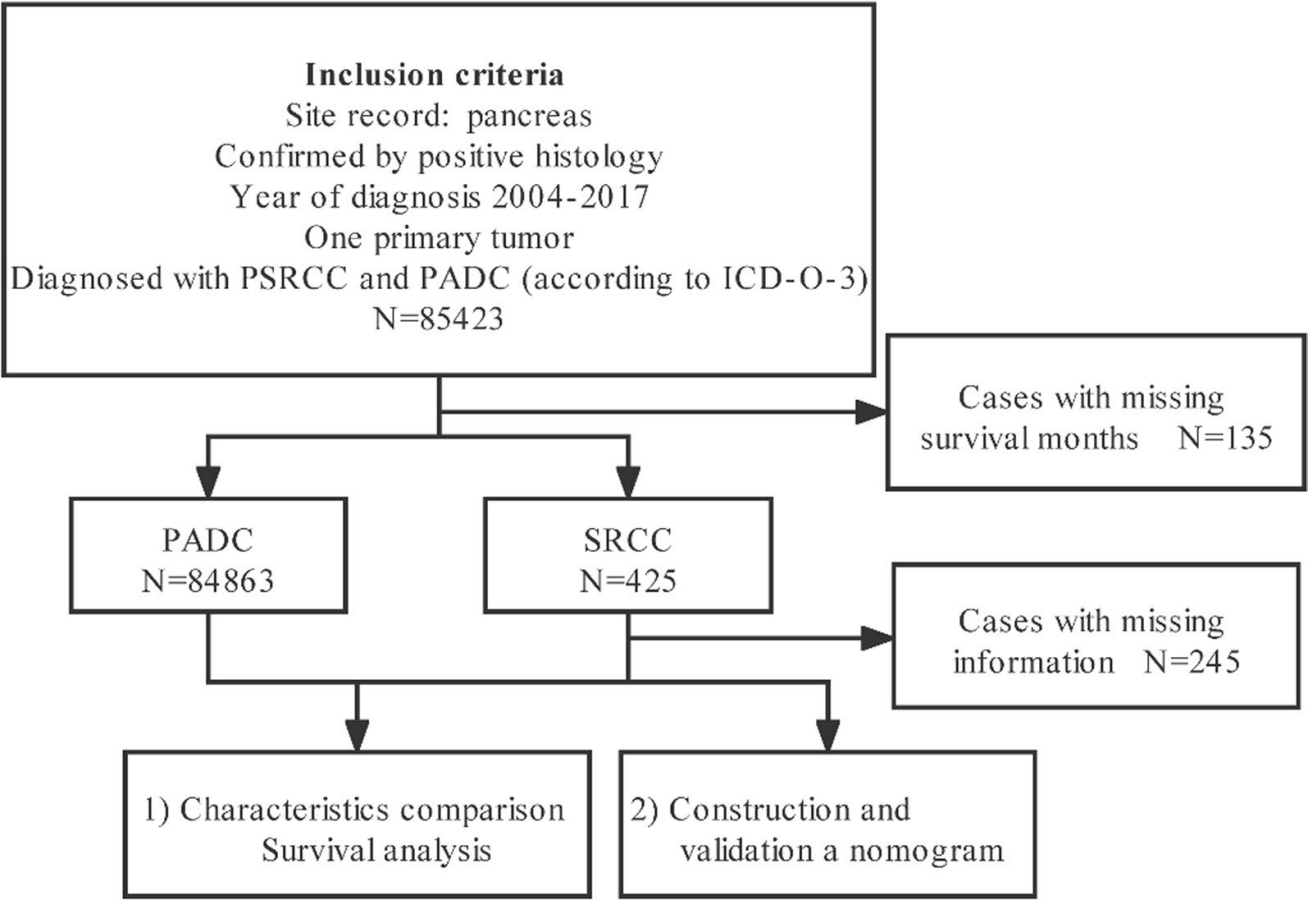
Patient selection flowchart

The SEER database collects incidence data of 18 population-based cancer registries and tumor clinicopathological information founding approximately 27.8% of the U.S. population. The SEER*Stat software (Surveillance Research Program, National Cancer Institute SEER*Stat software (www.seer.cancer.gov/seerstat, Version 8.3.9.) was used to extract all pathologically confirmed primary PSRCC and PDAC patients diagnosed from the SEER database between 2004 and 2017. The following variables were extracted: age at diagnosis, sex, race, marital status at diagnosis, primary tumor location, histology grade, AJCC TNM stage, T stage, N stage, M stage, chemotherapy, surgery, and radiotherapy, survival months, vital status, cancer-special survival. To assess the clinical characteristics of both as accurately as possible, we excluded only 135 patients who had no survival months. Finally, 84,863 patients with PDAC and 425 patients with PSRCC were enrolled in a comparative study of clinical characteristics and outcomes. When the independent prognostic factors of PSRCC were identified, incomplete clinical information was deleted, and eventually 180 patients were included in the establishment of the Cox regression model and the construction of nomogram. The specific flow chart of patient inclusion and exclusion is shown in Fig. 1.

### Statistics analysis

The annual age-adjusted incidence of PSRCC and PDAC were estimated using SEER*Stat software. Annual percentage changes (APCs) for assessing the changes of incidence were calculated by the Joinpoint Regression Program (Version 4.9.1.0. April, 2022; Statistical Research and Applications Branch, National Cancer Institute). Subsequently, we performed descriptive statistics on the demographic and tumor characteristics of the patients. Categorical variables were expressed in frequency and percentage, and using Pearson Chi-square test or Fisher exact test to compare the differences between groups. The survival curve was drawn by Kaplan–Meier method, and the differences between groups were compared by Log-rank test. Multivariate Cox regression models were used to correct confounding factors, such as: age at diagnosis, gender, primary tumor site, AJCC TNM stage, surgery, chemotherapy and radiotherapy, to determine the independent prognostic effect of PSRCC.

The independent predictors of OS in patients with PSRCC were determine by univariate and multivariate Cox proportional risk regression models. Based on significant prognostic factors in the multivariable analysis, we constructed a nomogram to predict 1-, 3-, and 5-year OS. The performance of the nomogram was evaluated by discrimination and calibration [18]. The discrimination was quantified by Harrell's Consistency index (C-index) and displayed with receiver operating characteristic (ROC) curve**.** The calibration was presented by calibration plot**.** The C-indexes and calibration plots for internal validation of the nomogram were calculated by the bootstrap analysis with 500 resample [19]. The clinical utility of nomogram and AJCC TNM stage are also measured by the DCA curve.

All statistical analysis and plotting were performed using R 4.0.4 (http://www.r-project.org/). R project’s packages, such as ‘survminer’, ‘survival’, ‘rms’ and ‘foreign’, were applied for multivariate Cox analysis and draw nomogram and calibration plot, ‘survivalROC’ was used for model validation and AUC analysis, and ‘ggDCA’ was used for DCA analysis. The statistical test was two-sided and P < 0.05 was regarded as statistically significant.

## Results

### Morbidity characteristics

**Fig. 2:**
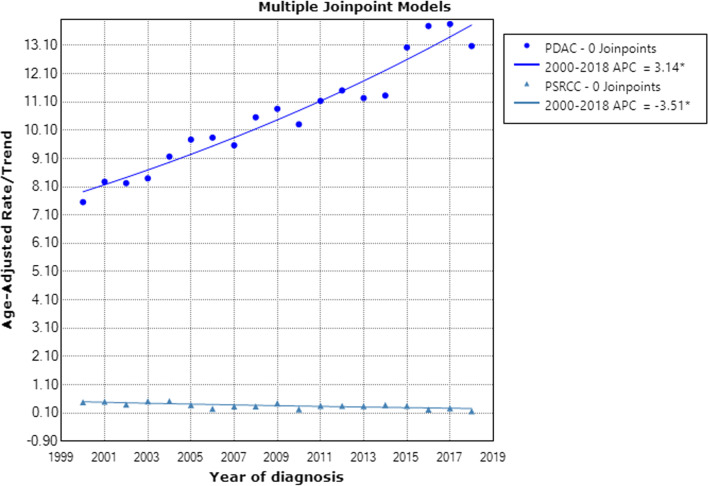
The age-adjusted incidence of PDAC and PSRCC patients between 2000 and 2018 from the SEER database

To describe the incidence of these two histological types, we selected patients with ductal adenocarcinoma of the pancreas and signet-ring cell carcinoma from the SEER database between 2000 and 2018. The results showed that overall incidence of PSRCC is much lower than that of PDAC (10.798 V.S. 0.349 per millions). And the incidence of PDAC has increased significantly over the past 18 years, whereas the incidence of PSRCC has decreased significantly (Fig. 2). APC was 3.1% (95% CI: 2.7–3.6) and -3.5% (95% CI: − 5.3% to 1.7%), respectively.

### Demographic and clinical characteristics

A total of 85,288 eligible patients including 425 PSRCC and 84,863 PDAC cases were identified in the study. The patients’ baseline features were demonstrated in Table 1. There were no statistically significant differences between the 2 comparison populations in demographic characteristics such as age at diagnosis, race, marital status at diagnosis, T stage, surgery and radiotherapy. The majority of patients in PDAC (79.0%) and PSRCC (83.1%) groups were white, but there was no remarkable difference in the proportion of race between the two groups (P = 0.219). Nevertheless, PSRCC was related to a worse grade with 38.6% of patients having grade III (vs. 14.9) and 1.9% of patients having grade IV disease (vs. 0.6%). In addition, PSRCC was associated with higher ratio of lymph node metastasis (38.8% vs. 35.3%, P = 0.004) and distant metastasis (51.6% vs. 62.4%, P < 0.001). In accordance with this consequence, patients with PSRCC were diagnosed at an advanced stage and were more likely to receive palliative chemotherapy than those with PDAC. Interestingly, PDAC is more likely to have a tumor in the head of the pancreas than signet ring cell histology (51.1% vs. 44.9%, P = 0.005).

**Table 1:** The clinical characters of the patients with pancreatic PSRCC and PDAC

Variable	Levels	PDAC (N = 84,863)	SRCC (N = 425)	P-value
Age at diagnosis	<= 70	50,220 (59.2%)	259 (60.9%)	0.461
	> 70	34,643 (40.8%)	166 (39.1%)	
Gender	Female	41,150 (48.5%)	175 (41.2%)	**0.003**
	Male	43,713 (51.5%)	250 (58.8%)	
Race	Black	10,624 (12.5%)	41 (9.6%)	0.219
	Other	7015 (8.3%)	30 (7.1%)	
	Unknown	204 (0.2%)	1 (0.2%)	
	White	67,020 (79.0%)	353 (83.1%)	
Marital status	Married	47,335 (55.8%)	241 (56.7%)	0.86
	Unknown	3552 (4.2%)	19 (4.5%)	
	Unmarried	33,976 (40.0%)	165 (38.8%)	
Grade	I	3479 (4.1%)	0 (0.0%)	**< 0.001**
	II	13,680 (16.1%)	23 (5.4%)	
	III	12,642 (14.9%)	164 (38.6%)	
	IV	471 (0.6%)	8 (1.9%)	
	Unknown	54,591 (64.3%)	230 (54.1%)	
Primary site	Body	10,959 (12.9)	48 (11.3)	**0.005**
	Head	43,324 (51.1)	191 (44.9)	
	Tail	11,267 (13.3)	63 (14.8)	
	Pancreatic duct	506 (0.6)	3 (0.7)	
	Islets of Langerhans	2 (0)	0 (0)	
	Other specified parts of pancreas	1298 (1.5)	3 (0.7)	
	Overlapping lesion of pancreas	6498 (7.7)	33 (7.8)	
	Pancreas, NOS	11,009 (13.0)	84 (19.8)	
TNM stage	I	5309 (6.3)	10 (2.4)	**< 0.001**
	II	22,439 (26.4)	98 (23.1)	
	III	8201 (9.7)	30 (7.1)	
	IV	43,747 (51.6)	265 (62.4)	
	Unknown	5167 (6.1)	22 (5.2)	
T stage	T0	488 (0.6)	2 (0.5)	0.127
	T1	2619 (3.1)	12 (2.8)	
	T2	16,377 (19.3)	62 (14.6)	
	T3	32,602 (38.4)	162 (38.1)	
	T4	16,563 (19.5)	90 (21.2)	
	Tx	15,826 (18.6)	96 (22.6)	
	Unknown	388 (0.5)	1 (0.2)	
N stage	N0	40,953 (48.3)	171 (40.2)	**0.004**
	N1	29,925 (35.3)	165 (38.8)	
	Nx	13,597 (16)	88 (20.7)	
	Unknown	388 (0.5)	1 (0.2)	
M stage	M0	37,992 (44.8)	146 (34.4)	**< 0.001**
	M1	43,747 (51.6)	265 (62.4)	
	Mx	2736 (3.2)	13 (3.1)	
	Unknown	388 (0.5)	1 (0.2)	
Surgery	No	67,385 (79.4%)	349 (82.1%)	0.31
	Unknown	399 (0.5%)	1 (0.2%)	
	Yes	17,079 (20.1%)	75 (17.6%)	
Radiotherapy	Yes	14,354 (16.9%)	57 (13.4%)	0.055
	No/unknown	70,509 (83.1%)	368 (86.6%)	
Chemotherapy	No/unknown	36,974 (43.6%)	220 (51.8%)	**0.001**
	Yes	47,889 (56.4%)	205 (48.2%)	

*P* < 0.05 was bolded, indicating that the difference was statistically significant

### Survival analyses

**Fig. 3:**
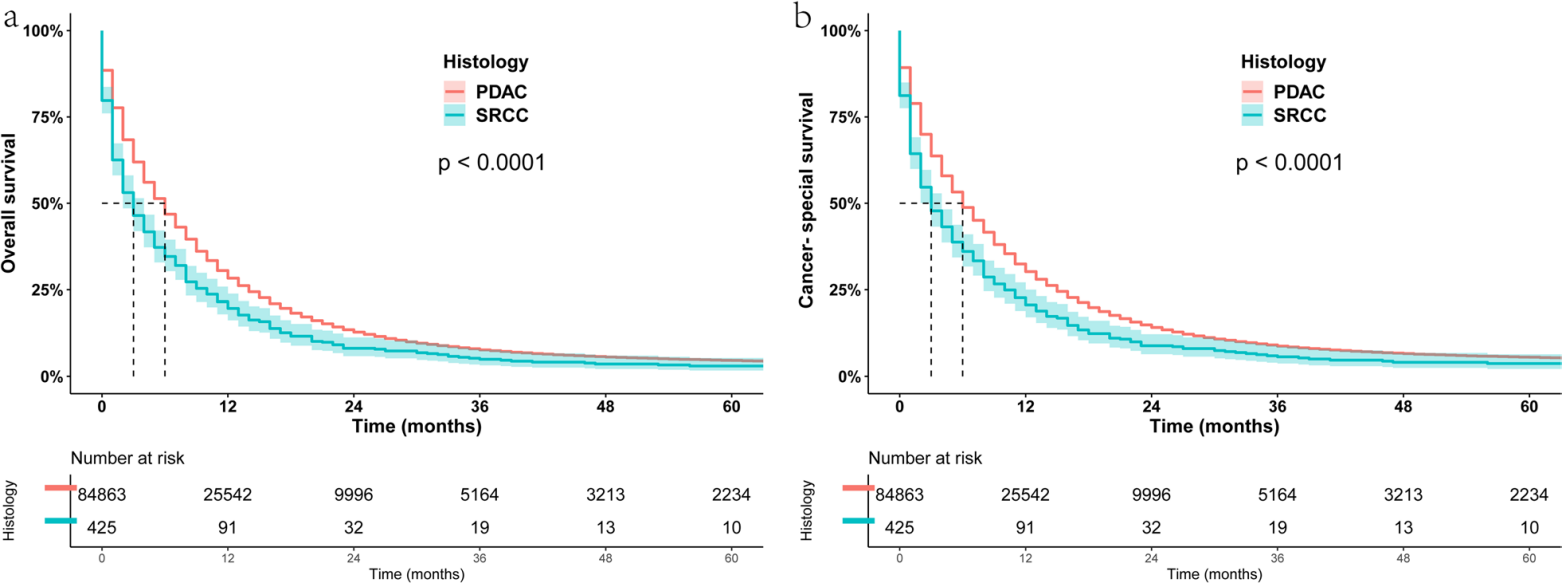
**a** Kaplan–Meier estimated overall survival for patients with PSRCC and PDAC. **b** Kaplan–Meier estimated cancer-special survival for patients with PSRCC and PDAC

**Fig. 4:**
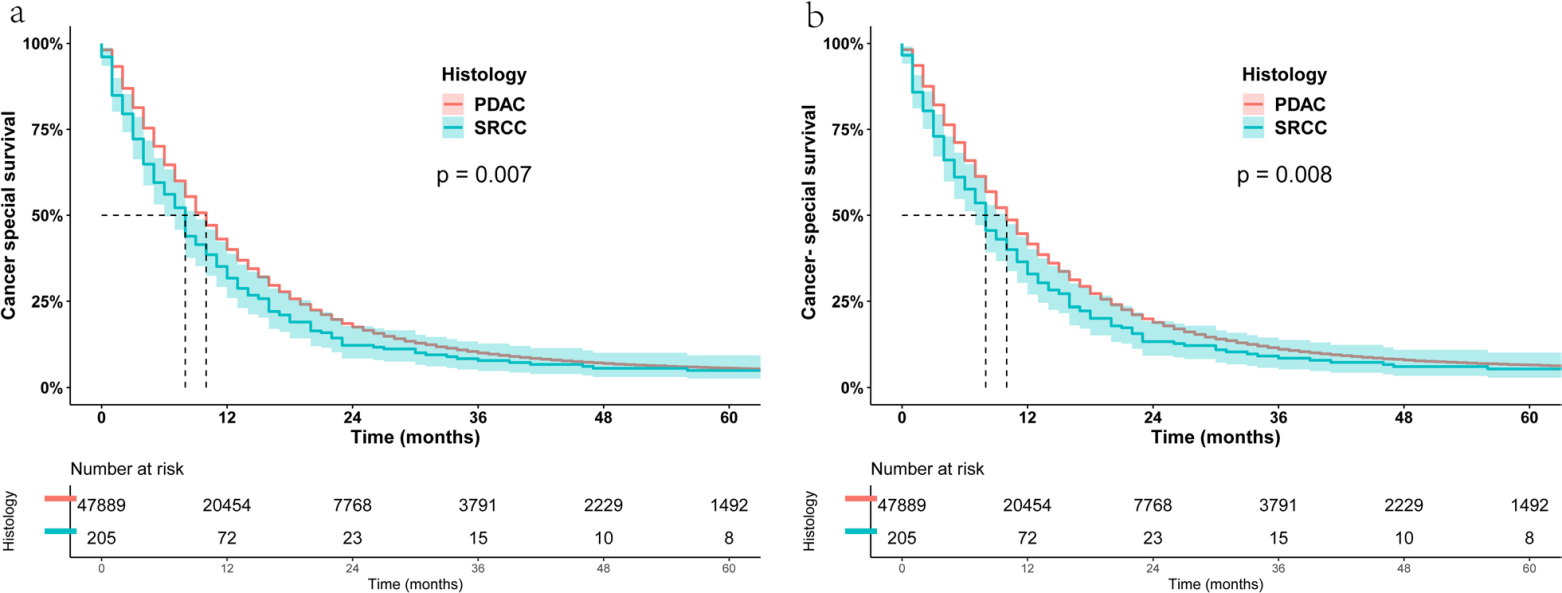
**a** Kaplan–Meier assessed the overall survival of PSRCC and PDAC patients receiving chemotherapy. **b** Kaplan–Meier estimated cancer-special survival of PSRCC and PDAC patients receiving chemotherapy

To analyze the survival difference between PSRCC and PDAC, we performed a KM survival curve analysis (Fig. 3a). The 1-, 3- and 5-year OS of PSRCC were 19.67%, 4.96% and 3.01%, respectively, while those of PDAC were 28.33%, 7.67% and 4.61%, respectively, with significant differences (P < 0.05). Similarly, PSRCC still had a worse prognosis than PDAC when comparing CSS between the two groups (Fig. 3b). Furthermore, we assessed the prognostic relationship between the two histological types in patients receiving chemotherapy and found that PSRCC continued to have a poor prognosis in patients receiving chemotherapy (Fig. 4a, b).

To determine whether signet ring cell histology was an independent predictor for pancreatic cancer, we adjusted for confounders using a Cox proportional risk model. The non-adjusted and adjusted models are demonstrated in Table 2. SRCC was considered an independent predictor of pancreatic cancer before adjusting for confounders. After adjustment for confounders, SRCC also remained an independent prognostic factor for pancreatic cancer, and patients with SRCC had a significantly higher risk of overall mortality rate compared with PDAC group (HR = 1.18, 95% CI: 1.07–1.30, P < 0.001)**.** Moreover, The SRCC patients had a higher risk of cancer-specific mortality (HR = 1.19, 95% CI = 1.08–1.31, P < 0.001).

**Table 2:** Unadjusted and adjusted Cox regression models

Outcomes	SRCC HR (95% CI)	P-value
*Overall mortality*		
Non-adjusted	1.34 (1.21–1.47)	< 0.001
Adjusted	1.18 (1.07–1.30)	< 0.001
*Cancer-specific mortality*		
Non-adjusted	1.35 (1.22–1.49)	< 0.001
Adjusted	1.19 (1.08–1.31)	< 0.001

### Construction and validation of nomogram

**Fig. 5:**
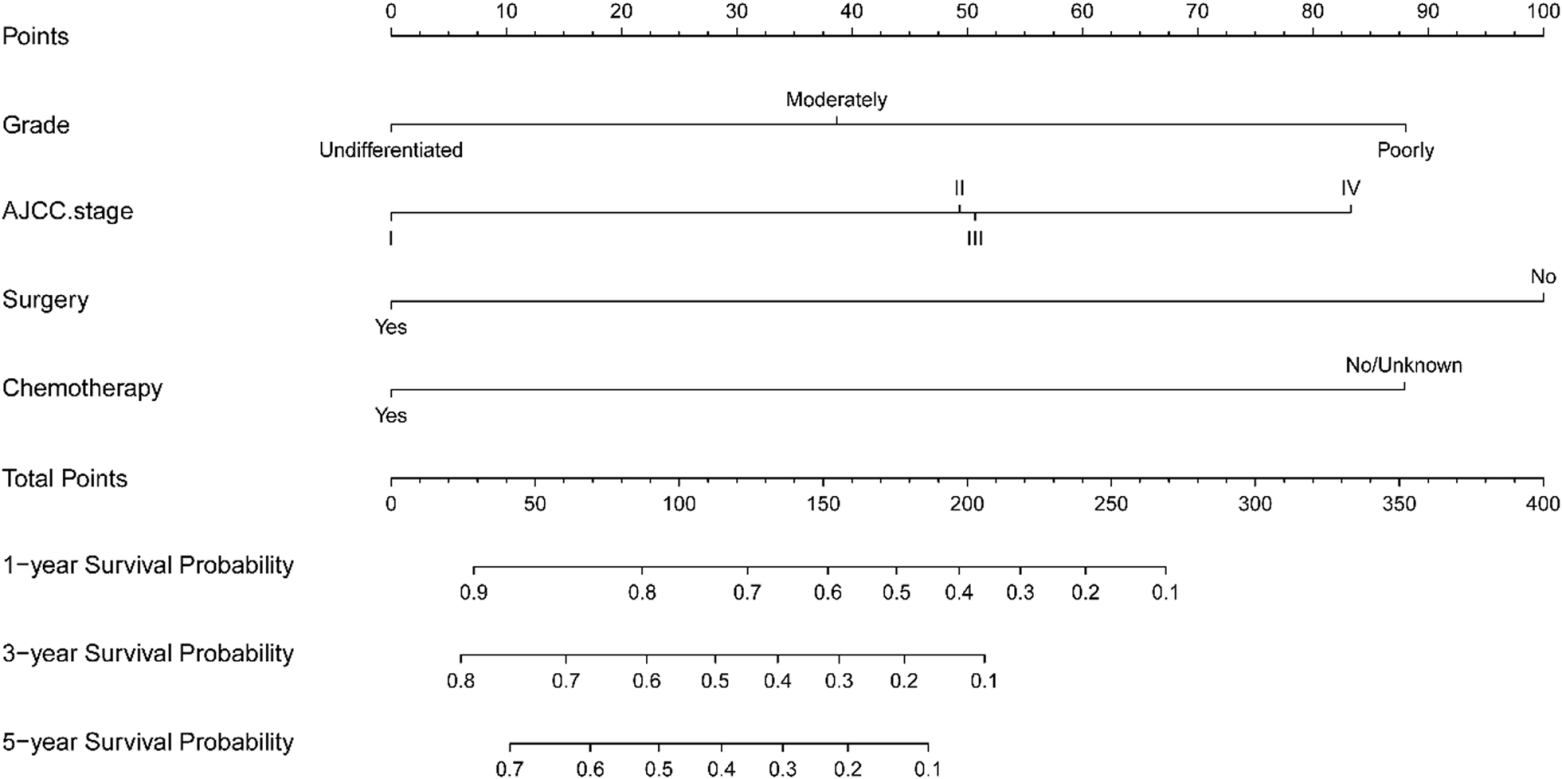
Nomogram predicting 1-, 3- and 5-year OS of PSRCC

Since PSRCC has worse biological behavior and prognosis than PDAC, we identified the prognostic factors that affect PSRCC by performing Cox regression analysis. The significant factors in univariate Cox regression were included in the multivariate analysis (Table 3). The results suggest that grade, AJCC TNM stage, surgery and chemotherapy have a significant impact on the prognosis. Based on this result, we established a nomogram to predict the OS of PSRCC patients (Fig. 5). By drawing a straight line on the point axis, each independent risk factor could correspond to a specific score. The total score reflects the sum of the scores of various factors, drawn downward from the straight line of the total score axis 1-, 3-, and 5-year corresponding to the predicted 1-, 3-, and 5-year OS, respectively.

**Table 3:** Univariate and multivariate analyses for OS of patients with PSRCC

Variables	Level	Overall (N = 180)	HR (95% CI)	P-value	HR (95% CI)	P-value
Age (median [IQR])		66.0 (59.0, 75.0)	1.02 (1–1.03)	0.017	1.01 (1–1.02)	0.1986
Race (%)	Black	15 (8.3)	Reference		/	
	Others	14 (7.8)	1.64 (0.77–3.53)	0.201	/	
	White	151 (83.9)	1.26 (0.71–2.24)	0.427	/	
Sex (%)	Female	72 (40.0)	Reference		/	
	Male	108 (60.0)	1.1 (0.81–1.5)	0.545	/	
Grade (%)	Moderately	22 (12.2)	Reference		Reference	
	Poorly	150 (83.3)	2.41 (1.43–4.08)	0.001	1.9 (1.11–3.25)	0.0195
	Undifferentiated	8 (4.4)	2.38 (0.98–5.79)	0.056	0.83 (0.29–2.42)	0.7361
Marital status (%)	Married	102 (56.7)	Reference			
	Unmarried	78 (43.3)	1.17 (0.86–1.59)	0.314	/	
Primary site (%)	Body	17 (9.4)	Reference		/	
	Head	107 (59.4)	0.8 (0.47–1.37)	0.423	/	
	Others	39 (21.7)	1.8 (1–3.24)	0.052	/	
	Tail	17 (9.4)	1.19 (0.59–2.39)	0.622	/	
AJCC stage (%)	I	7 (3.9)	Reference		Reference	
	II	71 (39.4)	1.27 (0.51–3.17)	0.606	1.91 (0.76–4.82)	0.1714
	III	18 (10.0)	2.2 (0.81–5.98)	0.121	1.95 (0.71–5.36)	0.1930
	IV	84 (46.7)	3.72 (1.5–9.27)	0.005	2.98 (1.16–7.65)	0.0232
Surgery (%)	No	111 (61.7)	Reference		Reference	
	Yes	69 (38.3)	0.27 (0.19–0.39)	< 0.001	0.27 (0.18–0.42)	< 0.001
Chemotherapy (%)	No/unknown	86 (47.8)	Reference		Reference	
	Yes	94 (52.2)	0.46 (0.34–0.63)	< 0.001	0.33 (0.22–0.48)	< 0.001
Radiation (%)	Yes	38 (21.1)	Reference		Reference	
	None/unknown	142 (78.9)	1.93 (1.32–2.82)	0.001	0.85 (0.54–1.35)	0.4887

**Fig. 6:**
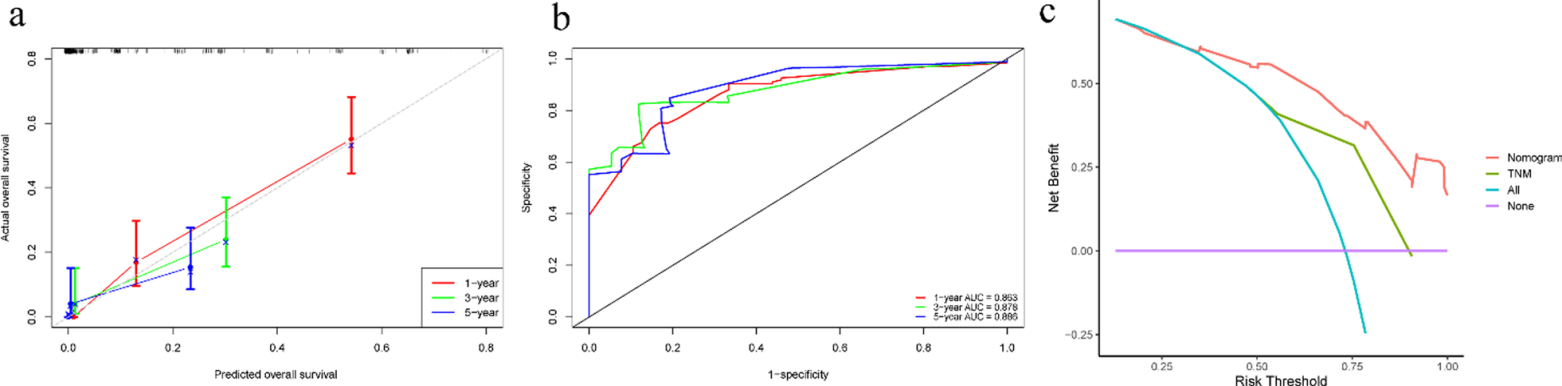
Performance of the nomogram for PSRCC patients. **a** tROC curves and AUC at 1-, 3-, and 5-year OS. **b** Calibration plot for predicting 1-, 3-, and 5-year OS. **c** DCA of the prognostic nomogram and AJCC TNM stage

Due to the rarity of PSRCC, the bootstrap analyses with 500 resample were operated for internal verification to ensure maximum use of data. Our model has good discriminative ability than AJCC stage, the C indexes of our nomogram and AJCC stage before calibration were 0.763 and 0.650, respectively, while the C-indexes after 500 samples were resampled were 0.750 and 0.646, respectively. Similarly, ROC curve analysis also indicated that the nomogram had good discrimination, with an area under the ROC curve (AUC) of 0.863, 0.876, and 0.886 for 1-, 3-, and 5-year OS, respectively (Fig. 6a). In addition, the resampled calibration plots show that there is good agreement between the 1-, 3-, and 5-year OS predicted by the nomogram and the actual observation results (Fig. 6b). Moreover, the DCA curve also demonstrated that our model had better clinical practicability than the traditional TNM stage (Fig. 6c).

## Discussion

PSRCC is a rare tumor whose clinical features and prognosis have not been adequately described. We conducted our study based on a population-based SEER database. The research proposed to compare the clinical characteristic and prognosis of PSRCC and PDAC. We found that compared with PDAC, PSRCC have a poorer histological grade, a higher ratio of lymph node and distant metastasis, and a poorer prognosis. In addition, we also found that PSRCC is an independent predictor of pancreatic adenocarcinoma. Therefore, we have developed a nomogram to manage PSRCC to provide convenience for clinicians in clinical decision-making.

### Rare but deadly

It is well known that primary SRCC is most frequently found in the stomach and PSRCC is extremely rare. Only three large studies have mentioned PSRCC. Two of these studies [20, 21] only compared the survival difference between PSRCC and other primary SRCC, and found that the prognosis of PSRCC was the worst. Patel et al. [3] collected data of patients diagnosed with PSRCC diagnosed from 1973 to 2013 in the SEER database, and found that the distant metastasis rate of SRCC was high, and only 3% were located in the pancreas at the time of diagnosis. Unfortunately, these three studies did not compare the incidence and clinical characteristics of patients with PSRCC and PDAC. In our study, PSRCC is a rare tumor whose incidence is decreasing year by year. Furthermore, we found that compared with PDAC patients, patients with PSRCC had a higher ratio of lymph node metastasis and distant metastasis, and a worse histological grade and prognosis. Moreover, multivariate Cox regression suggested that histology of SRCC is an independent prognostic factor for pancreatic cancer. In general, PSRCC has poorer histological behavior than PDAC and is of greater concern to clinicians.

### PSRCC may be sensitive to specific chemotherapeutic agents

Previously, SRCC seemed to be a tumor with low sensitivity to chemotherapy. However, Pernot’s study suggests that gastric SRCC may have particular sensitivity characteristics and prone to be more sensitive to taxane-based chemotherapy or antiangiogenic agents [22]. Similarly, Kim et al. [23] reported that taxane-based chemotherapy was connect with an 80% R0-related resection rate and median overall survival beyond 40 months in a limited group of patients who were diagnosed with gastroesophageal SRCC (n = 17). Taxane-based chemotherapy may be a potential therapy option for SRCC as the same pathologic type, despite being located at different sites. In addition, Radojkovic et al. [9] reported a case of that was treated with gemcitabine neoadjuvant chemotherapy for 3 months. Efficacy evaluation showed a good response to chemotherapy and radical surgery was performed. These data demonstrate to a certain extent that PSRCC is sensitive to specific chemotherapy agents. In our study, patients with PSRCC who received chemotherapy had significantly better outcomes than those who did not, which was confirmed by multivariate Cox regression, suggesting that chemotherapy was regarded as a protective predictor with HR of 0.50 (95% CI, 0.34–0.71; P < 0.001). This suggests that clinicians may need to consider the specific chemotherapeutic sensitivity of SRCC when selecting agents. Furthermore, multivariate Cox regression manifested that grade, AJCC stage and surgery were independent factors affecting the prognosis of PSRCC. Surgery has been shown to be a favorable factor for PSRCC, and this has been confirmed in the literature.

### Independent management tool for PSRCC

Nomogram includes various cancer-related risk factors and visually presents their impact on patient survival [24]. It is a common instrument to assess cancer patients condition of prognosis and personally predict the survival rate of patients [25]. So far, several nomograms for predicting the prognosis of pancreatic cancer have been established [26–29]. But our research shows that SRCC is an independent prognostic factor for pancreatic cancer. It will bring more heterogeneity if the two are managed uniformly. Therefore, we established a nomogram based on the results of multivariate Cox regression. In contrast, our nomogram has the following advantages. Firstly, this is the first predictive nomogram for PSRCC, which can provide a good tool for the management of rare PSRCC. Secondly, this nomogram includes common clinical factors, including surgery, chemotherapy, histological grade, primary site, and AJCC TNM stage, which is more personalized than traditional AJCC stage. Our nomograms can predict the time points of at 1-, 2-, and 5-year survival probability, making them easy to judge the prognosis, and they have better distinguishing ability and calibration ability. Finally, the current nomogram is not a single-center study. This model can be applied for individualized prognostic evaluation, and an effective diagnostic tool that may help make treatment-related decisions can be promoted.

### Limitations

The study had several limitations. Firstly, due to the lack of key information in the SEER database, including physical condition, surgical details, chemotherapy regimen and course, chemotherapy and surgical sequence, we are limited to draw more meaningful conclusions. In addition, previous studies have shown that endoscopic-guided tumor ablation combined with intraperitoneal plexus release is superior to intraperitoneal plexus release alone in terms of pain control and overall survival [30]. Information on non-surgical interventional therapy is also lacking in the SEER database, which deserves our attention in the future. Secondly, it should be noted that this research was retrospective, and data containing missing values were deleted during the construction of the nomogram, which may result in selection bias, whereas the strict validation method and large sample size can improve our accuracy. Finally, as most people in SEER database are white, our study and the nomogram may be more suitable for white people.

## Conclusion

In conclusion, PSRCC are a rare but fatal subtype of pancreatic cancer and a nomogram was firstly used to manage the OS of PSRCC, which is expected to help pathologists and oncologists design clinical decisions for this rare pathological subtype.

## Data Availability

The data cited in this study is publicly achievable in the Surveillance Epidemiology and End Results Program at https://seer.cancer.gov/.
